# Nursing Research on the United Nations Sustainable Development Goals—A Bibliometric Analysis

**DOI:** 10.1111/jan.16953

**Published:** 2025-04-07

**Authors:** Christopher Holmberg, Linda Ahlstrom

**Affiliations:** ^1^ Institute of Health and Care Sciences University of Gothenburg Gothenburg Sweden; ^2^ Department of Psychotic Disorders Sahlgrenska University Hospital, Region Västra Götaland Gothenburg Sweden; ^3^ Department of Orthopaedics Sahlgrenska University Hospital, Region Västra Götaland Gothenburg Sweden

**Keywords:** evidence gaps, gender equity, global health, nursing research, patient care, sustainability, sustainable development

## Abstract

**Aim:**

The aim of this study is to map nursing publications on the United Nations Sustainable Development Goals (SDG) in Web of Science, highlighting trends, key contributors, and central research themes to identify potential areas for future research.

**Background:**

The globally‐spanning 2030 Agenda promotes sustainable development using research technology and scientific innovation. However, research data availability is a challenge. By conducting big data analyses, using all available nursing research literature indexed in the Web of Science database (Core Collection) pertaining to this field, aid in understanding and advancing this area.

**Methods:**

This study adopts a cross‐sectional descriptive bibliometric study design.

**Results:**

The search yielded 131 publications, comprising 116 articles (89%) and 15 review articles (11%). This can be compared to adjacent disciplines such as Internal General Medicine (*n* = 360), Nutrition/Dietetics (*n* = 171), and Paediatrics (*n* = 152). The leading countries in publication output were the United States, Australia, and the United Kingdom. Among the included publications, only eight SDGs were addressed: SDG3 (Good Health and Well‐Being), SDG13 (Climate Action), SDG4 (Quality Education), SDG5 (Gender Equality), SDG6 (Clean Water and Sanitation), SDG16 (Peace, Justice, and Strong Institutions), SDG1 (No Poverty), and SDG9 (Industry, Innovation, and Infrastructure).

**Conclusions:**

The findings indicate a scarcity of articles in nursing publications focusing on the SDGs, suggesting insufficient evidence of nursing's contributions to these goals—particularly beyond SDG3.

**Implications:**

This study provides a comprehensive bibliometric review and analysis of existing nursing publications on the SDGs. The results offer valuable insights for future research areas related to the SDGs, particularly for nursing scholars, clinicians, managers, and policymakers concerned with the underrepresentation of nursing publications. To address this gap and advance both the SDGs and quality patient care, action plans should be developed to integrate the SDGs into daily nursing practice.

No Patient or Public Contribution. This study was a bibliometric analysis.

## 1 Introduction

From the era of Florence Nightingale up to the present day, the environment has been recognised to play a crucial role in improving the patient's disease processes (Kiang and Behne 2021). Florence Nightingale implemented sustainable practices in healthcare settings by improving sanitation and hygiene standards, which had a significant impact on patient outcomes (Dossey et al. 2019). However, when the environment becomes contaminated or polluted, it can have detrimental effects on both short‐ and long‐term health outcomes (Fuller et al. 2022). From a nursing perspective, factors such as pollution, climate change, and the increase in antibiotic resistance due to large‐scale animal industries all influence human health and healthcare systems (World Health Organization 2023), but also underscore the need for nurses to be proactive.

The Sustainable Development Goals (SDGs) of the United Nations (UN) are highly relevant to clinical challenges that nurses encounter as they pertain to both the more dramatic national public health emergencies as well as everyday issues such as leadership and shared governance (World Health Organization 2021, 2023). The 2030 Agenda for Sustainable Development was a 15‐year plan agreed to by all UN member states which came into effect in 2016 (United Nationals Department of Economic and Social Affairs 2015). It was a global initiative aimed at fostering collective efforts to realise peace, prosperity, and well‐being for all, and protecting our planet by the year 2030 (Sachs 2012). Comprising 17 SDGs (Table 1), these goals address social, economic, and environmental determinants of health. The agenda includes 169 associated targets focused on five themes: people, planet, peace, prosperity, and partnership (United Nationals Department of Economic and Social Affairs 2015). These goals build upon the earlier UN Millennium Development Goals that were in place from 2000 to 2015 (Rosa et al. 2021).

**TABLE 1:** The United Nations Sustainable Development Goals (SDGs).

Goal	Description	Goal	Description
1	No Poverty. End poverty in all its forms everywhere	10	Reduced Inequalities. Reduce inequality within and among countries
2	Zero Hunger. End hunger, achieve food security and improved nutrition and promote sustainable agriculture	11	Sustainable Cities and Communities. Make cities and human settlements inclusive, safe, resilient and sustainable
3	Good Health and Well‐Being. Ensure healthy lives and promote well‐being for all at all ages	12	Responsible Consumption and Production. Ensure sustainable consumption and production patterns
4	Quality Education. Ensure inclusive and equitable quality education and promote lifelong learning opportunities for all	13	Climate Action. Take urgent action to combat climate change and its impacts
5	Gender Equality. Achieve gender equality and empower all women and girls	14	Life Below Water. Conserve and sustainably use the oceans, seas and marine resources for sustainable development
6	Clean Water and Sanitation. Ensure availability and sustainable management of water and sanitation for all	15	Life on Land. Protect, restore and promote sustainable use of terrestrial ecosystems, sustainably manage forests, combat desertification, and halt and reverse land degradation and halt biodiversity loss
7	Affordable and Clean Energy. Ensure access to affordable, reliable, sustainable and modern energy for all	16	Peace, Justice and Strong Institutions. Promote peaceful and inclusive societies for sustainable development, provide access to justice for all, and build effective, accountable and inclusive institutions at all levels
8	Decent Work and Economic Growth. Promote sustained, inclusive, and sustainable economic growth, full and productive employment and decent work for all	17	Partnerships for the Goals. Strengthen the means of implementation and revitalise the global partnership for sustainable development
9	Industry, Innovation, and Infrastructure. Build resilient infrastructure, promote inclusive and sustainable industrialization and foster innovation		

### 1.1 A Central Aspect of the 2030 Agenda Emphasises Planetary Health and Climate Action

Health systems consume substantial amounts of resources; focusing on sustainable practices can thus both enhance the quality of care while reducing waste production and emissions (Kiang and Behne 2021). While nursing and nursing scholars traditionally have focused on the social and economic determinants of health, the urgency highlighted by key nursing organisations such as the International Council of Nurses (ICN) compels nurses, as the largest healthcare workforce, to integrate the SDG framework into their actions (International Council of Nurses, 2024a, 2024b), indicating a growing recognition of the importance of environmental factors in influencing health outcomes. This shift in focus reflects an evolving understanding of the multifaceted nature of health and highlights the need for interdisciplinary collaboration to address emerging challenges in healthcare. Nurses are central in addressing the effects of climate change, as is demonstrated with the relevance of the Sustainable Development Goal 3 (Good Health and Well‐Being) as well as the importance of nursing education in environmental awareness aligned with Goal 4 (Quality Education) (Lilienfeld et al. 2018; Rosa et al. 2019). The literature on nursing and related healthcare demonstrates a growing recognition of the SDGs in connection to the foundational principles and future evolution of the nursing profession (Rosa et al. 2019; World Health Organization 2021).

The 2030 Agenda for sustainable development presents a data‐driven concept of governance and highlights the challenge of substantially increasing the availability of research data (Indana and Pahlevi 2023). This article therefore investigates the extent to which current research explores the impact of the SDGs in the nursing discipline. Bibliometric analysis has been used in nursing to analyse country‐wide publication patterns and research performance (Holmberg 2024; Nicoll et al. 2018). To our knowledge, only one previous bibliometric analysis has included aspects specifically related to sustainability within the health sector and the role of nurses in this process (Luque‐Alcaraz et al. 2022). Consequently, the present study was designed to address this gap. The aim of this study is to map nursing publications on the United Nations Sustainable Development Goals (SDG) in Web of Science, highlighting trends, key contributors, and central research themes, to identify potential areas for future research. Specifically, what are the quantity, characteristics, research collaborations, and research themes of nursing publications related to SDGs, and how have these aspects changed over time?

## 2 Methods

### 2.1 Study Design

The study adhered to the Preferred Reporting Items for Bibliometric Analysis (PRIBA), which is a modification of the PRISMA statement that accounts for the unique characteristics of bibliometric studies (Koo and Lin 2023). See Table S1 for the checklist. In doing so, the Scientific procedures and rationales for systematic literature reviews protocol (SPAR‐4‐SLR) was used to structure the bibliometric study in an orderly, rigorous, and transparent way (Paul et al. 2021). See Table 2 for an overview. The protocol provided us with a pre‐established set of structured elements and guided the assessment, arrangement, and assembling of bibliometric data for the analysis. Bibliometric analyses are suitable when the intention is to quantitatively assess the characteristics of scientific publications within a specific field or topic (Baraibar‐Diez et al. 2020).

**TABLE 2:** The SPAR‐4‐SLR protocol, as recommended by (Paul et al. 2021).

Assembling	**Identification** *Domain*: Nursing research, specifically focusing on the United Nations Sustainable Development Goals. *Research question*: Research performance and Intellectual structure. *Source type*: All article types. *Source quality*: Web of Science
	**Acquisition** *Search mechanism and material acquisition*: Web of Science Core Collection. *Search period*: 1 January 2016—31 December 2023. *Search keywords*: Sustainable development goals, SDGs, Agenda 2030. *Total number of articles returned from the search*: From search string (*N* = 23,794). Filtering to those only indexed as Nursing (*n* = 135) and excluding early access articles (*n* = 131)
Arranging	**Organisation** *Organising codes*: Article titles, article citations (global and local), author names, journal titles, author keywords, KeyWords Plus
	***Purification*** *Article type excluded* (and total number for each type of exclusion): Articles indexed as “early access” (*n* = 4) *Article type included* (and total number of articles included): Articles, *n* = 116 (e.g., research papers, brief communications, technical notes) and review articles, *n* = 15 (i.e., literature reviews)
Assessing	**Evaluation** **Analysis method**: Bibliometric and content analyses **Agenda proposal method**: Thematic (implications and future research)
	**Reporting** **Reporting conventions**: Figures (networks and graphs), tables and words (descriptions and narratives) **Limitations**: Only publications in English

### 2.2 Assembling

The first step in the process involves identification and collecting publications for analysis, known as assembling. The dataset was obtained from the Web of Science Core Collection (Clarivate Inc.) on 28 January 2024. The selection of Web of Science was based on its status as a comprehensive and curated database aggregator, offering reliable bibliometric information, including a broad range of metadata parameters (Gusenbauer 2022; Holmberg 2023).

The following search string was created to identify publications aligning with the study aim. TS = (“SDGs” OR “sustainable development goal” OR “sustainable development goals” OR “agenda 2030” OR “2030 agenda”). “TS” denotes topic term searches, and it uses the articles' abstracts, titles, and keywords as search fields. Since the SDGs were enacted in 2016, the filter was set to publications published between the year 2016 up until 2023 to ascertain full calendar years.

### 2.3 Arranging

The subsequent phase, organisation and purification entails the systematic arrangement and enhancement of the articles based on inclusion and exclusion criteria. Various data elements, including journal title, author name, publication title, country of affiliation, total publications (TP), and total citations (TC), were employed as coding parameters to categorise the publication search data. These codes streamlined the process of organising and scrutinising the data in a structured and systematic fashion (Aria and Cuccurullo 2017).

### 2.4 Assessing

The final phase includes evaluation and reporting. Prior to initiating the primary analyses, an assessment was conducted on the quality of metadata associated with the acquired records. This evaluation included a thorough examination to identify any unattributed data, such as missing titles, and the removal of duplicate records. Furthermore, standardisation measures were applied to author and affiliated institute names to minimise potential confusion arising from variations in spelling, abbreviations, and initials.

To comprehend the publication landscape, a combination of bibliometric and content analyses was employed (Donthu et al. 2021). While bibliometric analyses focus on bibliographic data, encompassing numeric elements like publication year and number of citations, quantitative content analyses were used to evaluate frequently used words and terms reflecting research themes and topics. To assess the most frequent of the SDGs in the material, the research area schema “Sustainable Development Goals,” inherent in the Web of Science database, was used. The schema algorithmically assigns one or more of the 16 SDGs to a record (SDG17 is not included).

Ethical considerations: given that the bibliometric approach solely relied on publicly accessible metadata from Web of Science, ethical approval was not needed.

## 3 Results

After having applied all the filters in the search, a total of 23,794 articles were identified. The number of articles that met the inclusion criteria within the field of nursing, focusing on the United Nations Sustainable Development Goals, was *n* = 131, of which 116 (89%) were articles and 15 (11%) were review articles. This can be compared to related disciplines within health care, such as Internal General Medicine (*n* = 360), Nutrition/Dietetics (*n* = 171), Paediatrics (*n* = 152), Obstetrics/Gynaecology (*n* = 149). The largest research area overall, in terms of number of publications related to the SDGs, was Environmental sciences (*n* = 7610).

The 131 articles within Nursing were published in 52 different journals. The three most common journals to publish in were in order, International Nursing Review (*n* = 13), Midwifery (*n* = 10), and Journal of Nursing Scholarship (*n* = 7).

**FIGURE 1:**
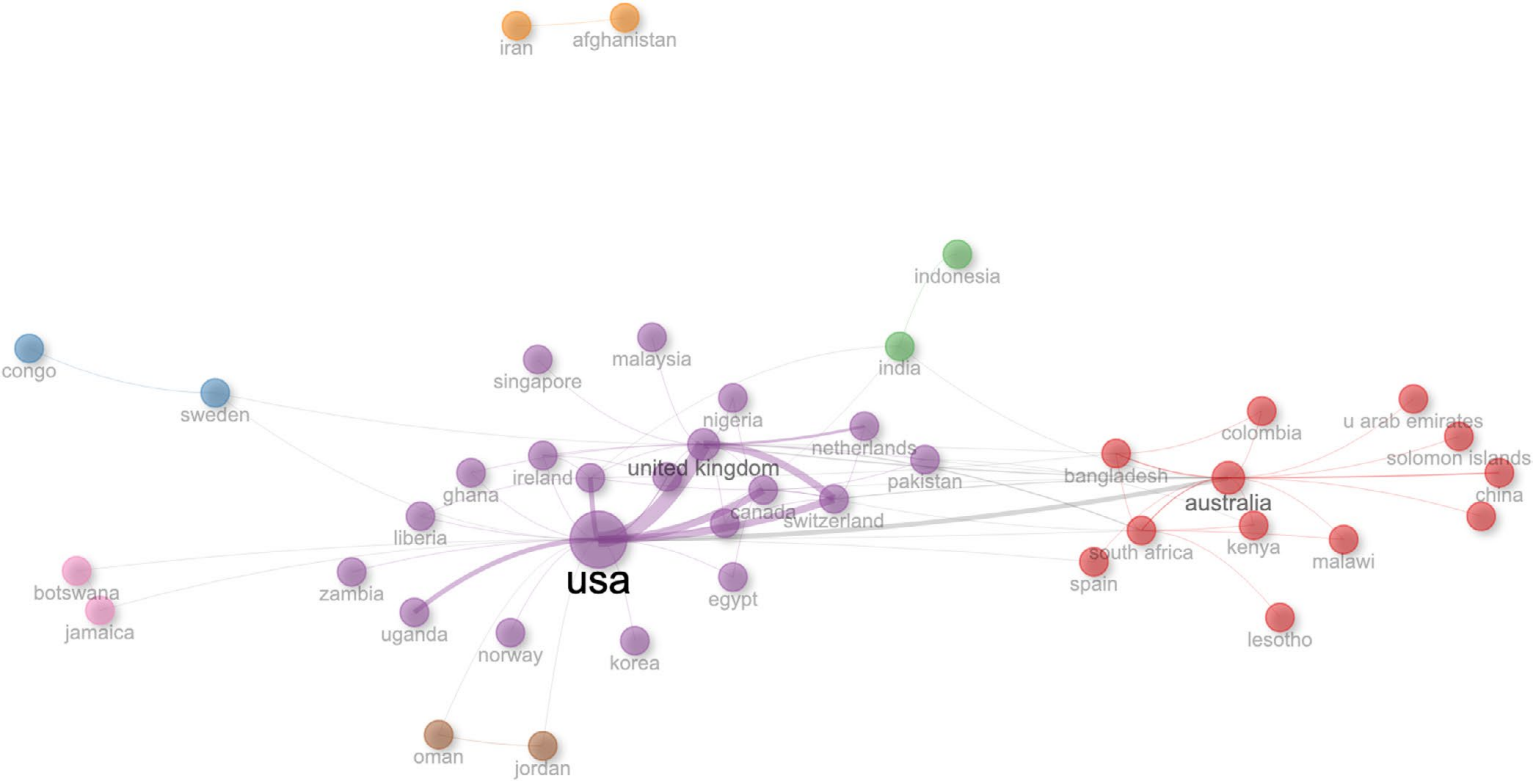
Country collaborations.

The countries with the most publications were the United States, US (*n* = 174), Australia (*n* = 68), and the United Kingdom UK (*n* = 38). International co‐authorships accounted for nearly 47% (46.56) of all publications. See Figure 1. The most common collaborations involved first authors from the US, often with one or more co‐authors from the UK (*n* = 7), Switzerland (*n* = 5), and Canada (*n* = 5). The most prolific authors were, in order, Dr. William Rosa (*n* = 12), Dr. Stephanie Ferguson (*n* = 5), and Dr. Michele Upvall (*n* = 5). All three authors have professional backgrounds in nursing. The institutes most frequently cited as author affiliations were the University of Pennsylvania in the United States (*n* = 14), University of Wollongong in Australia (*n* = 13), and the Memorial Sloan Kettering Cancer Center in the United States (*n* = 12).

**FIGURE 2:**
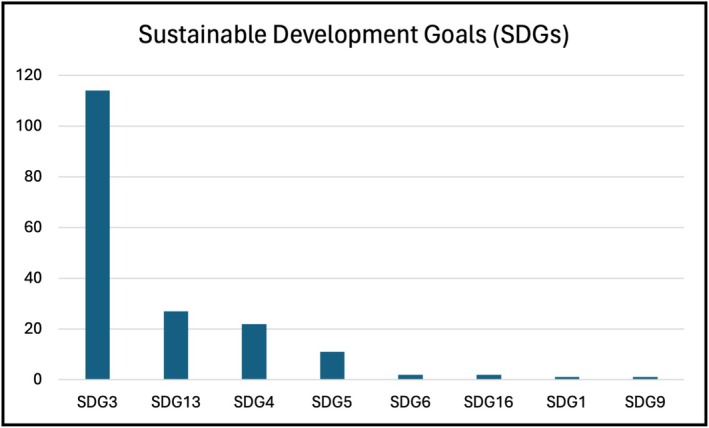
The Sustainable Development Goals (SDGs) applicable to the included articles (*n* = 131).

Using the research area schema for the SDGs inherent in Web of Science, as seen in Figure 2, most (*n* = 114) of the articles corresponded to SDG3 (Good Health and Well‐Being), followed by SDG13 (*n* = 27) (Climate Action), SDG4 (*n* = 22) (Quality Education), and SDG5 (*n* = 11) (Gender Equality). Only a few articles corresponded to SDG6 (*n* = 2) (Clean Water and Sanitation), SDG16 (*n* = 2) (Peace, Justice and Strong Institutions), SDG1 (*n* = 1) (No Poverty), and SDG9 (*n* = 1) (Industry, Innovation and Infrastructure) respectively. This means that articles pertaining to SDG2 (Zero Hunger), SDG7 (Affordable and Clean Energy), SDG8 (Decent Work and Economic Growth), SDG10 (Reduced Inequalities), SDG11 (Sustainable Cities and Communities), SDG12 (Responsible Consumption and Production), SDG14 (Life Below Water), and SDG15 (Life on Land) were absent in the material.

In total, the articles were cited 871 times (739 without self‐citations), meaning that the average number of citations per article was 6.65. The three most cited articles (average citation) were all literature reviews that presented factors that challenged or facilitated the achievement of the SDGs within different areas of the clinical nursing profession as well as the academic discipline of nursing (Bvumbwe and Mtshali 2018; Kurth 2017; Meleis 2016). The review by Bvumbwe and Mtshali (2018), cited 68 times, focused on educational factors and the need for more reforms in Sub‐Saharan Africa to augment the capacity of educators and mentors, the responsiveness of curricula, the strength of regulatory frameworks, and the availability of infrastructure and resources. The review by Meleis (2016), cited 54 times, focused on interprofessional education for health professionals to address the fundamental and most persistent barriers to forming equitable healthcare teams, which were identified as the consistent narrative of medical privilege and centrism. The article by Kurth (2017), cited 50 times, was a more general call to action within nursing, to recognise that nurses are instrumental in achieving the SDGs that align with the planetary health framework, focusing on environmental sustainability and human well‐being. Kurth (2017) furthermore stressed that nurses contribute to resilient healthcare systems in their capacity as trusted leaders and healthcare providers, while also acting as advocates and change agents impacting the surrounding world in relation to sustainable development.

### 3.1 Topics

**FIGURE 3:**
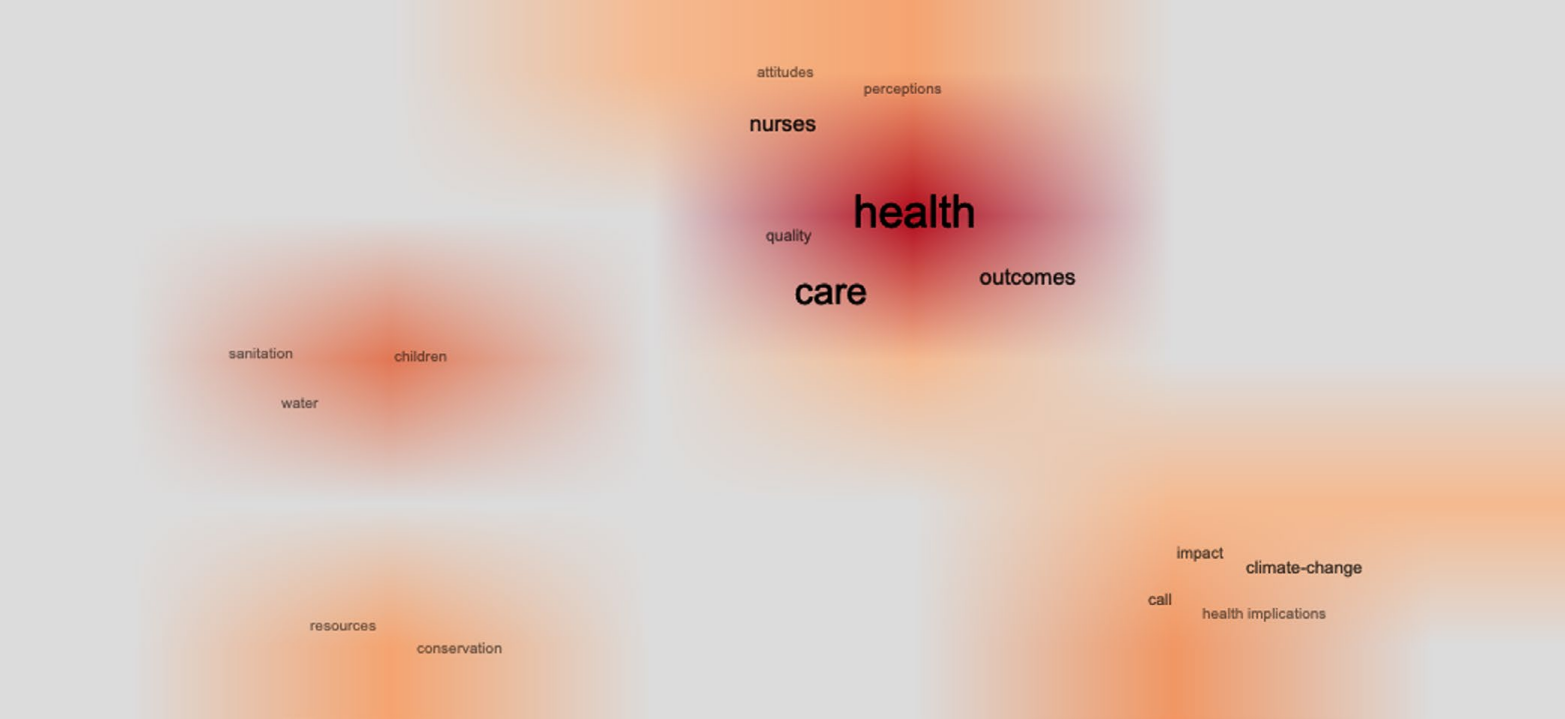
Co‐occurrence cluster network illustrated as an overlay based on KeyWords Plus.

Commonly co‐occurring words in the articles' keywords illustrate clusters of certain frequently connected topic areas. In doing so, four clusters (including one sub cluster) became visible, as illustrated in Figure 3.

*Cluster 1* consists of the commonly co‐occurring words “health,” “care,” “outcomes,” and “quality.” It reflects research related to the health and care outcomes achieved through various nursing interventions. Various aspects such as healthcare quality, patient satisfaction, health promotion, disease prevention, and management of health conditions are explored. This cluster is particularly related to SDG3. Adjacent to *Cluster 1*, a sub cluster could be visible, consisting of the commonly co‐occurring words “nurses,” “attitudes,” and “perceptions,” is centred around studying the attitudes and perceptions of nurses towards different aspects of their profession, healthcare systems and patient care. Understanding nurses' attitudes and perceptions is essential for enhancing their role in achieving sustainable development goals related to healthcare access, quality and equity. Research in this cluster delved into topics such as job satisfaction, burnout, professional identity, and perceptions of healthcare policies. *Cluster 2*, consisting of the commonly co‐occurring words “children,” “sanitation” and “water,” focuses on issues related to children's health, particularly in the context of sanitation and access to clean water. Research in this cluster pertained to SDG3 and SDG6. It examined the impact of poor sanitation and water quality on child health outcomes and explored nursing and midwifery interventions intended at improving access to essential services. *Cluster 3*, consisting of the commonly co‐occurring words “climate change,” “call,” “impact” and “health implications,” addresses the impact of climate change on healthcare systems and the importance of, and role of nurses in responding to related challenges. It involves research pertaining to SDG3 and SDG13, focusing on climate change adaptation strategies, disaster preparedness, and mitigation efforts within healthcare settings. *Cluster 4*, consisting of the commonly co‐occurring words “conservation” and “resources,” focuses on exploring the role of nurses in conservation efforts and resource management, reflecting the ecological dimension of sustainable development. It pertains to SDG5 and encompasses research on sustainable healthcare practices, including energy conservation, waste management, and efficient resource utilisation.

### 3.2 Trend Topics

**FIGURE 4:**
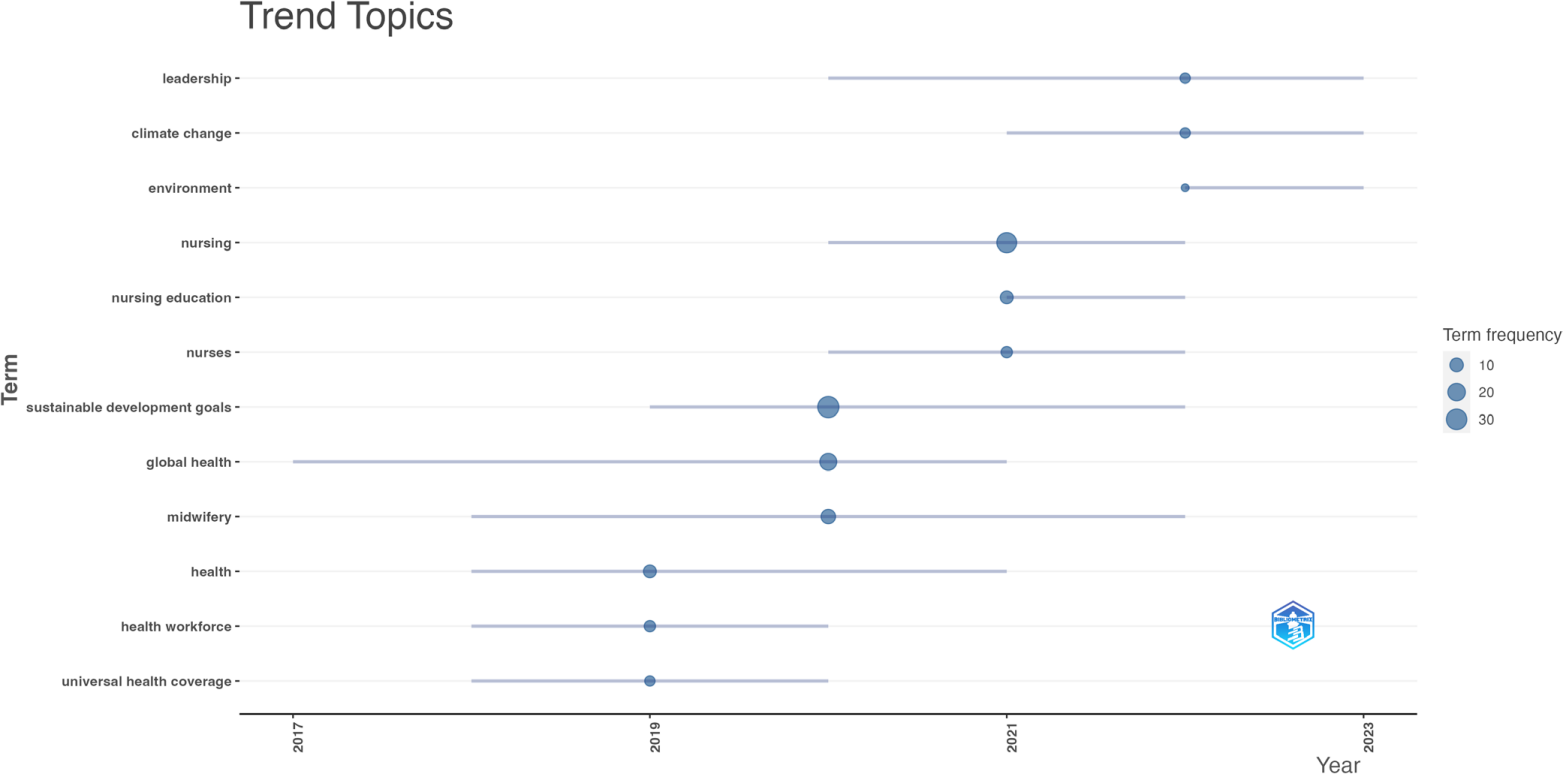
Trending topics across different years.

Analysing publication trends reveals varying common topics over different years. Between 2019 and 2020, prevalent terms included “health workforce,” “global health,” “midwifery” and “universal health coverage.” See Figure 4. This suggests a global outlook within the nursing literature regarding the Sustainable Development Goals (SDGs), with particular emphasis on the nursing workforce, including midwives. Notably, this aligns with 2020 being designated the Year of the Nurse and Midwife by the World Health Organisation. In contrast, from 2021 to 2022, common topics shifted to “nursing education,” “leadership,” “climate change” and “environment.” This indicates an increased emphasis on ecological dimensions of the SDGs, specifically climate change and environmental concerns, alongside recognition of nurses' roles as leaders and educators.

## 4 Discussion

When mapping nursing publications on the SDGs, we found that there were relatively few articles on the SDGs in nursing, as compared to that of other disciplines such as specific branches in Medicine that had more publications (i.e., internal medicine and paediatrics). Surprisingly, even nutrition/dietetics, with typically fewer clinical roles than nursing, showed a higher volume of publications. These findings align with previous literature reviews on the topic. One review, including 22 articles, found that a majority of publications consisted of editorial pieces or discussion papers (Osingada and Porta 2020). The other, based on 35 articles, only found four empirical articles (Fields et al. 2021). This is worrying as it is suggested that the dearth of research and scholarship accounts for the underrepresentation of nurses in policy and decision‐making processes (Ajuebor et al. 2019). This is echoed by some of the articles included in the present study that underscore that despite being the largest healthcare profession, nursing has been described as inadequately represented in policy and decision making in general, particularly when compared with medicine (Lamb et al. 2020; Rosa et al. 2019). There is a general scarcity of studies focused on nurses and environmentally sustainable health systems (Osingada and Porta 2020). Discrepancies exist between nursing interventions and research targeting environmentally sustainable health systems including nurses (Álvarez‐Nieto et al. 2022).

On a positive note, nursing students are exhibiting a growing favourable outlook on incorporating sustainability and climate change topics into their nursing curriculum. They acknowledge the significance of education on sustainability and the influence of climate change on health, advocating for formal training in environmental literacy. It is imperative to capitalise on this optimistic shift in nursing students' perspectives by seamlessly integrating these competencies into nursing curricula (Álvarez‐Nieto et al. 2022). Among the included articles, several underscored the necessity of incorporating SDGs content into both undergraduate and graduate nursing curricula. Nursing education and professional development programs are essential for equipping nurses with the knowledge, skills, and competencies needed to meet evolving healthcare challenges and contribute effectively to achieving the SDGs. Our research aligns with these goals, as common topics shifted to nursing education, leadership, climate change, and environment. Nurses are obligated to strategize their endeavours, interventions, clinical activities and scholarly pursuits methodically and intentionally in alignment with supporting and advocating for the SDGs. The efficacy of nurses' contributions to the SDGs will remain suboptimal if nursing fails to recognise the indispensable role of theoretical frameworks in shaping our perspectives and actions within the global landscape. Theory serves as a conduit for communication, empowerment, innovation, perseverance, cultural inheritance, and professional evolution. It constitutes a fundamental guiding force for our collective journey forward. Realising these goals, fostering a world characterised by compassion and well‐being, rooted in the scholarly insights and empirical foundations laid by our ancestors, while also paving the way for future generations to actively engage in transformative endeavours. Nursing theory embodies the continuum both in the present and extending into the post‐2030 era (Rosa et al. 2020).

The results indicate that only eight of the SDGs were covered by the included publications: SDG3 (Good Health and Well‐Being), SDG13 (Climate Action), SDG4 (Quality Education), SDG5 (Gender Equality), SDG6 (Clean Water and Sanitation), SDG16 (Peace, Justice, and Strong Institutions), SDG1 (No Poverty) and SDG9 (Industry, Innovation, and Infrastructure). This needs to be addressed by the nursing community. The nursing profession, including midwives, is regarded as trusted professionals in the public's eyes, and their collective voice is needed if we are to progress the SDG agenda and reorient services. Nursing plays a crucial role in promoting health and equity, ensuring access to quality healthcare for all, which aligns with SDG3. Nurses are at the forefront of providing essential healthcare services, addressing health disparities, and advocating for the health needs of diverse populations, thus contributing directly to the achievement of SDG 3. Additionally, SDG4—Quality Education emphasises the importance of lifelong learning and capacity building. Education in sustainability and raising awareness among nurses are considered essential pillars to reach the SDGs. Therefore, nurses play a pivotal role in driving change within the current health system towards environmental sustainability through research and integrated projects (Lilienfeld et al. 2018). Nursing is predominantly a female‐dominated profession. In Sweden, in 2022, 90% of the registered nurses were women (The Swedish National Board of Health and Welfare 2023), and as such, the profession plays a substantial role in advancing gender equality and women's empowerment, as outlined in SDG5. By promoting equal opportunities, supporting women's leadership roles in healthcare, and addressing gender‐based discrimination, nursing contributes to the broader agenda of gender equality.

The SDGs offer both theoretical groundwork and practical routes towards achieving a world of equity and social justice, leaving no one behind. It's crucial to develop concrete plans that translate these goals into local, measurable, and sustained actions within specific clinical settings. Sigma's advocacy at the UN serves as a prime example of the kind of enduring partnership that enhances the visibility and prominence of nurses (Sensor et al. 2021). Nurses can contribute to sustainability efforts within healthcare settings by advocating for environmentally friendly practices, reducing waste, and promoting resource efficiency, as the four clusters in our results showed, particularly cluster 2, focusing on environmental and ecological aspects such as sanitation and water resources. This aligns with SDG13—Climate Action, as nurses play a crucial role in ensuring that healthcare services are delivered in a sustainable and environmentally conscious manner.

The results also show that the United States was the most prolific country in nursing publications pertaining to the SDGs. Most top contributing countries can be labelled as high‐ or middle‐income countries, which has been found in previous reviews (Osingada and Porta 2020). The lack of research publications from low‐income nations is challenging as the SDGs suggest a global scale to the solutions and innovations that can help address the problems associated with the SDGs. Positively, nursing professionals often engage in global health initiatives and partnerships, working collaboratively to address health challenges beyond national borders. Our results show collaboration between the United States and Australia, and this should be further emphasised and recognised as significant in nursing projects. This aligns with SDG17—Partnerships for the Goals, as nurses contribute to cross‐sectoral collaborations, knowledge sharing, and capacity building efforts aimed at achieving the SDGs collectively. Organisational aspects and working with and towards policymakers, and nurse managers and educators need to offer robust support to staff nurses, ensuring they possess the requisite blend of competencies, expertise, and attitudes pertaining to preventive care and public health. This entails a concentrated emphasis on care coordination, patient‐centric approaches, quality enhancement, and proficiency in data analytics (Oerther and Rosa 2020). However, scholars highlight that the need for increased research capacity is not the sole area requiring attention. Globally, there is a pressing demand for more nurses to bolster the profession and advance towards the attainment of SDG3 and the universal health coverage target (Benton et al. 2020). This corresponds with a thematic cluster identified in our findings, which underscores the significance of workforce and staffing issues. An empirical investigation delving into global health workforce metrics, concentrating on 74 nations with the highest infant mortality rates, revealed a notable deficit in healthcare personnel, with nurses comprising a substantial proportion. This shortage falls significantly below the levels recommended by the WHO to effectively address the objectives outlined in SDG3 (Pozo‐Martin et al. 2017).

Ultimately, the time to act is now. Even before the COVID‐19 pandemic, global progress towards achieving the SDGs by 2030 was insufficient. The pandemic, coupled with ongoing crises, has further impeded and, in some cases, reversed countries' advancement towards these global goals (United Nationals Department of Economic and Social Affairs 2023).

Nurses have a unique opportunity and responsibility to lead the way towards a more sustainable, equitable, and healthier future for all. By harnessing their collective expertise, passion, and commitment to social justice, nurses can play a transformative role in advancing the SDGs agenda and creating positive change in communities around the world.

### 4.1 Strengths and Limitations

There are certain strengths and limitations associated with this study that should be addressed.

Compared to previous literature reviews on this topic in nursing, this study included a substantially larger dataset, encompassing 131 publications and related metadata. This constitutes a more robust and comprehensive insight into the general research landscape. However, the quality of bibliometric data depends on its accuracy and completeness. Since we did not screen or analyse the articles in full text, we lack contextual information about the content and quality of individual publications. The Web of Science was used to identify articles. Using a more specialised search engine like PubMed might have provided slightly different results. However, when comparing the same databases across different platforms, such as Medline via PubMed or Medline via Web of Science, the search results tend to be similar (Gusenbauer 2022). Also, Web of Science was selected deliberately because it is an interdisciplinary database aggregator, which helped us identify articles focusing on the SDGs in nursing across different fields. In the operationalisation of searches, we did not consider adding or replacing existing terms with more general terms like “sustainability” or “environment.” While this might have resulted in more articles, we prioritised precision and thus focused on articles explicitly using iterations of SDGs or Agenda 2030 in their title or abstract. However, besides searching in article titles and abstracts, article keywords were also used for identification purposes. Keywords are reliable indicators for estimating subject coverage as they are available across databases (Gusenbauer 2022).

## 5 Conclusions

The results reveal a scarcity of articles in nursing explicitly focusing on the SDGs. There is a shortage of evidence to demonstrate how nursing is contributing to the SDGs, particularly outside of SDG3 (Good Health and Well‐Being). This presents both a gap and an opportunity for future nursing research. Nurses and midwives should recognise how the health of individuals and the community is impacted by the larger factors in our environment. The SDGs are of utmost importance for nursing as they provide a comprehensive framework for addressing global health challenges, promoting equity and sustainability, and guiding nursing practice, education, future research and advocacy efforts towards a healthier and more equitable world.

### 5.1 Implications

There is a lack of experimental data and policies on achieving or maintaining environmentally sustainable health care systems, indicating that nurses have an important role and should be consulted and included in decision‐making policies regarding sustainability in the health care systems (Luque‐Alcaraz et al. 2022).

Tailored action plans can be developed at various levels of proposed engagement to address the specific local requirements across different settings suggestible initiated by nurse managers and down the line implemented. In many healthcare organisations, while the top governance may have obtained certification for SDG, a significant challenge lies in ensuring that this vision permeates throughout all levels of the organisation, particularly among operational staff such as nurses. Despite the overarching commitment to sustainability at the organisational level, there is often a gap in communication and engagement that prevents frontline healthcare workers from fully understanding and embracing these SDGs. This lack of involvement can hinder the effective implementation of sustainable practices and initiatives within the healthcare setting. Therefore, it is imperative for healthcare leaders to prioritise communication and engagement strategies that empower all employees, including nurses, to understand the importance of SDGs and actively contribute to their realisation. By fostering a culture of sustainability that is inclusive and participatory, healthcare organisations can better align their operational practices with their SDGs, ultimately advancing both environmental stewardship and the delivery of quality patient care. Top‐down decisions on new routines need to be perceived as clear, fair, and well‐communicated. Particularly important areas include information about how to work with SDGs daily within nursing.

## Conflicts of Interest

The authors declare no conflicts of interest.

## Supporting information


**Table S1.** The Preferred Reporting Items for Bibliometric Analysis (PRIBA) checklist. As proposed by Koo, M., & Lin, S. C. (2023).

## Data Availability

All data supporting the findings of this study can be found in the Web of Science Core Collection. The dataset used in the analyses is available from the corresponding author upon reasonable request.
