# Supplementary material for: Nursing Research on the United Nations Sustainable Development Goals—A Bibliometric Analysis
Source: J Adv Nurs. 2025 Apr 7;81(12):8256–65. doi: 10.1111/jan.16953 (PMC12623695; doi:10.1111/jan.16953)
Supplement: Supplementary file 1 — Table S1. The Preferred Reporting Items for Bibliometric Analysis (PRIBA) checklist. As proposed by Koo, M., & Lin, S. C. (2023). [file JAN-81-8256-s001.docx]

| **Supplementary Table 1**. The Preferred Reporting Items for Bibliometric Analysis (PRIBA) checklist. As proposed by Koo, M., & Lin, S. C. (2023). | | |
| --- | --- | --- |
| **Section and topic** | **Item** | **Proposed item to be used in bibliometric research** |
| Title | 1a | Identify the study is a bibliometric analysis. |
| Title | 1b | Indicate the coverage period. |
|  |  |  |
| Abstract | 2a | Provide an explicit statement of objective(s). |
| Abstract | 2b | Specify the data sources.2cSpecify the coverage period. |
| Abstract | 2c | Specify the coverage period.2dProvide results for main outcomes. |
| Abstract | 2d | Provide results for main outcomes |
| Abstract | 2e | Provide an overall interpretation of the results and implications. |
|  |  |  |
| Introduction | 3 | Describe the rationale for the study |
| Introduction | 4 | Provide an explicit statement of objective(s). |
|  |  |  |
| Methods | 5a | Specific the database(s) or other data sources searched. |
| Methods | 5b | Describe the characteristics of the data source. |
| Methods | 5c | Specify the date when the search was conducted. |
| Methods | 6 | Specify the inclusion and exclusion criteria, such as language, article types, and coverage period. |
| Methods | 7 | Specify the search strategy and keywords used. Bibliometric indicators. |
| Methods | 8 | Describe the bibliometric indicators used. |
| Methods | 9 | Specify the software package(s) used and the settings selected. |
|  |  |  |
| Results | 10 | Describe the results of the search and selection processes, and use a flow diagram if necessary. |
| Results | 11 | Describe the results of bibliometric indicators, including quantity, performance, and structural elements. |
| Results | 12 | Prepare figures with an adequate resolution for online and print readability |
|  |  |  |
| Discussion | 13 | Summarize key results with reference to study objective(s). |
| Discussion | 14 | Discuss any limitations and impact of potential bias. |
| Discussion | 15 | Interpret the results in the context of background knowledge. |
|  |  |  |
| Other information | 16 | Describe the sources of financial or non-financial support and the role of funders or sponsors. |
| Other information | 17 | Declare any competing interests of the author(s). |
| Other information | 18 | Specify if data are publicly available and the route of access. |
